# Climate change impacts on population growth across a species’ range differ due to nonlinear responses of populations to climate and variation in rates of climate change

**DOI:** 10.1371/journal.pone.0247290

**Published:** 2021-03-03

**Authors:** Allison M. Louthan, William Morris

**Affiliations:** 1 Biology, Duke University, Durham, North Carolina, United States of America; 2 Division of Biology, Kansas State University, Manhattan, Kansas, United States of America; University of Michigan, UNITED STATES

## Abstract

Impacts of climate change can differ substantially across species’ geographic ranges, and impacts on a given population can be difficult to predict accurately. A commonly used approximation for the impacts of climate change on the population growth rate is the product of local changes in each climate variable (which may differ among populations) and the sensitivity (the derivative of the population growth rate with respect to that climate variable), summed across climate variables. However, this approximation may not be accurate for predicting changes in population growth rate across geographic ranges, because the sensitivities to climate variables or the rate of climate change may differ among populations. In addition, while this approximation assumes a linear response of population growth rate to climate, population growth rate is typically a nonlinear function of climate variables. Here, we use climate-driven integral projection models combined with projections of future climate to predict changes in population growth rate from 2008 to 2099 for an uncommon alpine plant species, *Douglasia alaskana*, in a rapidly warming location, southcentral Alaska USA. We dissect the causes of among-population variation in climate change impacts, including magnitude of climate change in each population and nonlinearities in population response to climate change. We show that much of the variation in climate change impacts across *D*. *alaskana’*s range arises from nonlinearities in population response to climate. Our results highlight the critical role of nonlinear responses to climate change impacts, suggesting that current responses to increases in temperature or changes in precipitation may not continue indefinitely under continued changes in climate. Further, our results suggest the degree of nonlinearity in climate responses and the shape of responses (e.g., convex or concave) can differ substantially across populations, such that populations may differ dramatically in responses to future climate even when their current responses are quite similar.

## Introduction

Effects of climate change on species’ geographic distributions are well-documented and widespread [1, 2]. However, the impact of climate change on individual populations within a species’ range can differ dramatically. In fact, climate-induced range shifts will only occur when climate change effects differ across a species’ range. For example, when populations located where conditions are cooler are able to persist or even grow under climate change, but populations located where conditions are warmer decline to extinction, a species’ range will shift to track its thermal tolerances. Accurately predicting climate change impacts on extant populations and on shifts in species’ ranges requires understanding the factors that generate variation in climate change impacts on population growth rate across a species’ range.

For terrestrial widespread species, all populations across the range are unlikely to experience equivalent rates of change in temperature and precipitation. In particular, species with wide latitudinal ranges are likely to experience faster rates of warming at their poleward limits than at their equatorward limits [3]. However, these latitudinal patterns in rates of warming can be complicated by topography, elevation, or buffering impacts of the ocean [4], as well as by changes in precipitation that may counter or exacerbate the negative effects of temperature increases. The variation in magnitude of climate change can contribute to variation in response to climate change among populations; for example, a recent review showed that population declines are more severe where average annual temperature changes are more substantial [5]. In addition to among-population differences in the magnitude of change in annual climate, the seasonal pattern of change in each climate variable may differ among populations [6].

Moreover, populations of the same species may differ in their intrinsic sensitivity to the same amount of change in a given climate variable [7–9]. One way to measure this climate sensitivity is to compute the derivative of the population growth rate with respect to a climate variable, evaluated at the current value of that variable. The difference in the rate of population growth between the future and current climates could then be approximated by multiplying the change in each climate variable by its climate sensitivity, and summing the products over all climate variables, a procedure akin to a so-called “life table response experiment” or LTRE [10, cf. 11]. Inherent in the LTRE approach is the assumption that the population growth rate changes linearly in response to a change in a climate variable. Even if the linear assumption were true, populations could differ in their sensitivities to climate variables (i.e., differ in the slope of the relationship between population growth and climate), due to life history differences among populations [12, 13]. Differences in (linear) sensitivities could contribute to among-population differences in climate change impacts. For example, low climate sensitivity may buffer some populations from changes in a given climate variable, while high sensitivity may amplify the impact of changes in the same climate variable in other populations.

**Fig 1:**
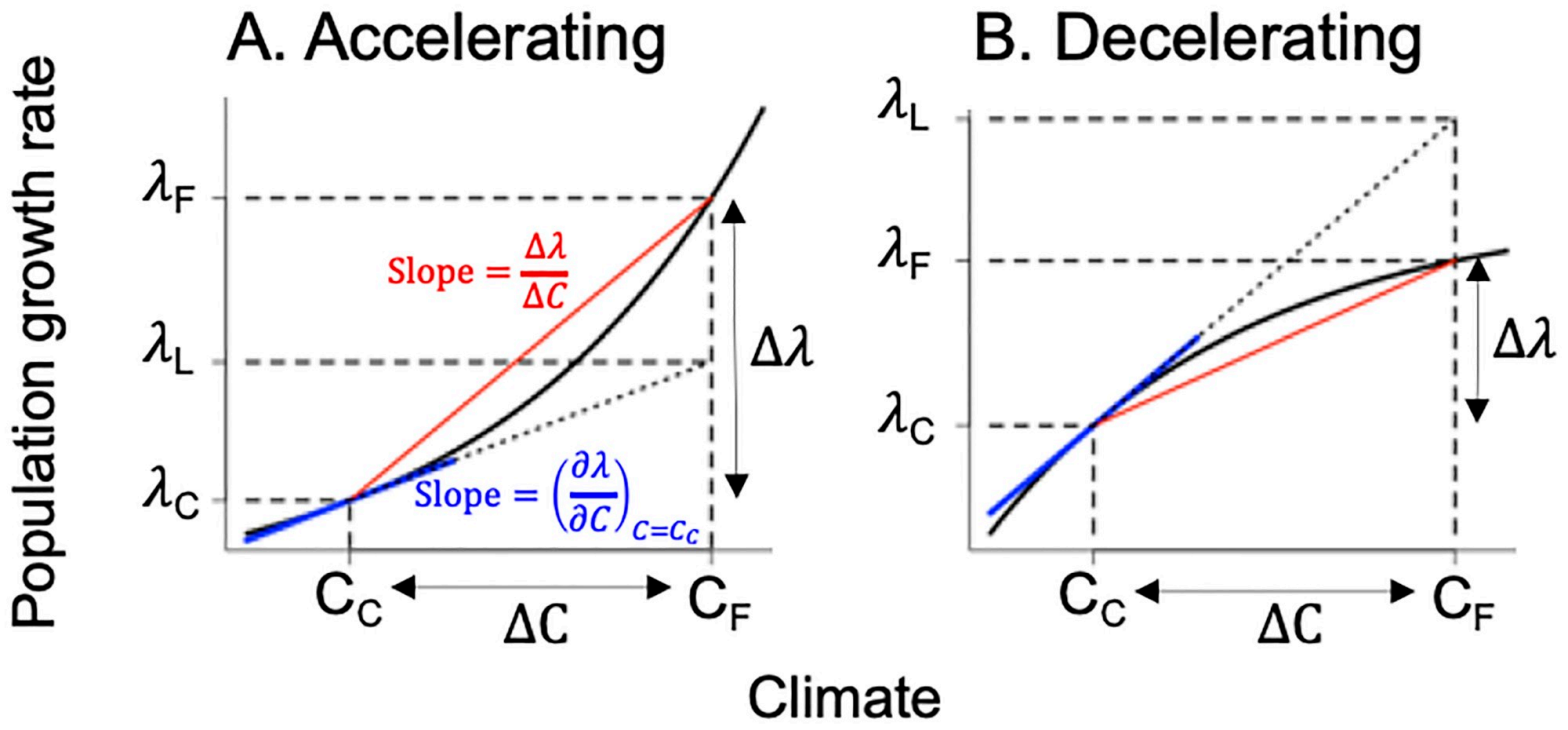
Non-linear effects of climate on population growth rate. Population growth rate is in general a non-linear function of climate (solid black lines), either: A. accelerating; or B. decelerating. λ_C_ and λ_F_ are the current and future growth rates at the current and future values of a climate variable; C_C_ represents current and C_F_ represents future climate. Blue lines show the slope of the population growth rate vs. climate evaluated at the current climate (i.e., “climate sensitivity”). The slope of the red line (“climate responsiveness”) is the ratio of the actual change in population growth Δλ to the actual change in climate ΔC (C_F_ -C_C_). λ_L_ is the linear projection of the future population growth rate using the sensitivity, which underestimates λ_F_ when population growth is an accelerating function of climate but overestimates λ_F_ when population growth is a decelerating function.

However, it is well known that the population growth rate is a non-linear function of the underlying vital rates [10, 14], and thus changes in climate variables may, if large enough, drive a change in the population growth rate that is substantially different than the linear approximation. In this case, the climate sensitivity may either underestimate (if population growth is an accelerating function of the climate variable; Fig 1A) or overestimate (if population growth is a decelerating function of the climate variable; Fig 1B) the true population response to climate change (hereafter, “climate responsiveness”, measured as the change in the population growth rate divided by the actual change in climate). Among-population differences in climate responsiveness will shape the responses to different magnitudes of climate change across a species’ range. Thus, to gauge the consequences of climate change on populations across species’ geographic ranges accurately, we must account for the true pattern of population growth responses to climate in different populations.

Here, we use an uncommon boreal plant species, *Douglasia alaskana* (Primulaceae), to test how the pattern of response to changes in climate variables and different magnitudes of climate change combine to influence population growth rate across much of a species’ range. *Douglasia alaskana* has a moderately-sized geographic range (southcentral Alaska), but has high habitat specificity (alpine scree fields) and small population sizes, satisfying two of the three components of rarity (habitat restriction and small local populations) described by ref. [15]. Rates of climate change are likely to be large but quite variable among populations, because the species occurs in one of the fastest warming regions in the world (the boreal zone), but some populations are coastal and others inland. We construct climate-driven integral projection models [16] for five populations using observed climate responses coupled with realistic projections of future climate. We then use these models to determine whether impacts of climate change on population growth rate are driven by the magnitude of climate change in each population v. climate responsiveness, and how nonlinearities in population responses to multiple components of climate (e.g., summer v. winter temperatures) contribute to variation in climate change effects across the range.

## Methods

**Fig 2:**
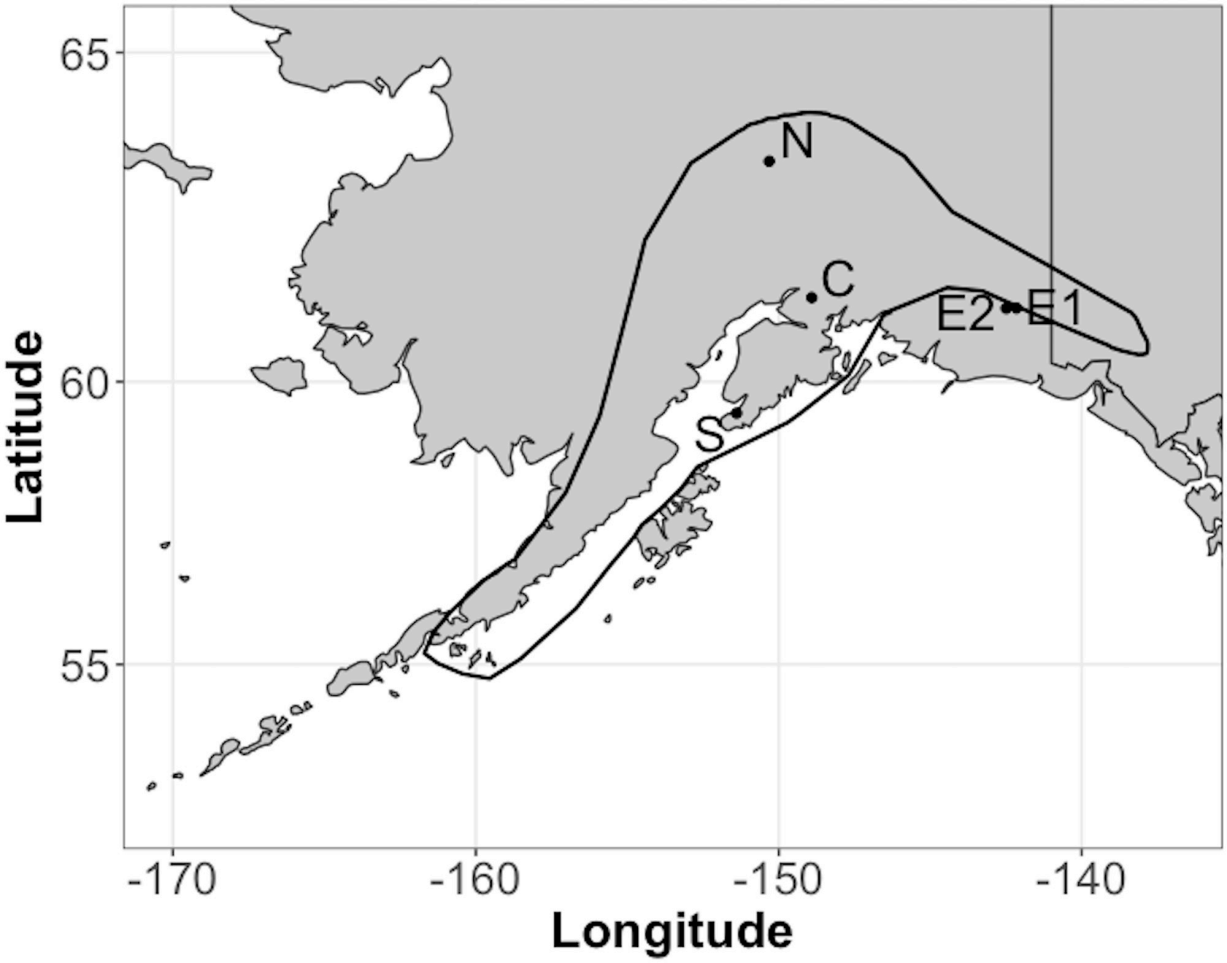
Range of *Douglasia alaskana* and location of study. Map of southcentral Alaska in grey, with the approximate range of *D*. *alaskana* indicated by the black polygon [17]. Populations used in this work are indicated by dots, and the labels of populations correspond to those of other figures. The E2 and E1 populations are offset from one another to allow readability.

*Douglasia alaskana* is a perennial alpine plant with 1–3 rosettes and fruits presented on short (<15 cm) peduncles. It occurs in alpine scree on ridgelines and mountaintops in southcentral Alaska, USA (Fig 2). Populations are small, ranging from 4–300 individuals in all populations we have found. The species is uncommon, with a very small fraction of seemingly suitable habitat occupied. Seeds are dropped within 15 cm of the parent plant into unvegetated scree fields (A. Louthan, pers. obs.). The species is partially semelparous, with most individuals (775 of 987 in our study) dying after reproducing. Median size of reproducing plans is 3.04 cm^3^, minimum size of reproducing plants is 0.32.

To assess *D*. *alaskana’*s response to climate change, we conducted a demographic study in populations spanning most of the range [17]. We then regressed vital rates against the climate conditions observed during our study. We used these regressions to construct population-specific climate-driven integral projection models (IPMs). We used projections of future climate from Global Climate Models (GCMs) to predict changes in population growth rate in each population. Finally, we used a decomposition approach to quantify the relative importance of different aspects of climate in eliciting climate change responses. We describe each step in more detail below.

To quantify response of *D*. *alaskana* to climate, we conducted demographic censuses in five populations (Fig 2) over one annual transition per population. In 2016, we marked and mapped 56 and 196 individuals in 2 populations (C and S in Fig 2), measuring size (sum of basal area x height of all rosettes; S1 Appendix) and number of fruits of each individual. We returned in 2017 to score survival and measure size and number of fruits. In 2017–2018, we repeated this procedure on 3 additional populations, with 218, 309, and 383 individuals marked and mapped in the three populations in 2017. All together, across the 5 populations for the 2016–2018 period, we obtained data from 1162 individuals, including seedlings. We estimated 5 size-dependent vital rates from these data: annual survival, mean size after one year of growth, variance in size after one year of growth, probability of fruiting, and number of fruits given fruiting. In four of the populations, we also estimated seedlings (in the second survey) per fruit (in the first survey) by counting all seedlings within a 15 cm radius in the second survey around individuals that fruited in the first survey. Our C populations were located in Chugach State Park, S in Kachemak Bay State Park, N in Denali National Park, and E1 and E2 in Wrangell-St. Elias National Park and Preserve, and we obtained permits from each of these entities.

We used field-collected soil temperature data and climate projections from global climate models (GCMs) to fit vital rate functions for the 2016–2018 period, as well as to construct IPMs and project future population growth rate. In all analyses, climate data were summarized by month, the timescale of the available future climate projections. Years were defined by the August 1 to July 31 interval, determined by the timing of the demographic censuses. For example, to estimate temperature over the 2017–2018 interval, we used data from August 1, 2017 to July 31, 2018. To obtain soil temperature data, we buried 1 or 2 small temperature loggers (iButtons; www.maximintegrated.com/en/products/ibutton/ibuttons/index.cfm) ~5 cm underground in each population. We used the iButton data to obtain mean monthly soil temperature over the 2016–2017 or 2017–2018 time period in each population. We obtained current precipitation values from 5 GCMs for the 2015–2018 time period for each population [18], hereafter, “SNAP” data. We used 2015–2018 soil temperature and precipitation data to fit climate-vital rate relationships over the 2016–2018 period (S1 Appendix). We obtained future precipitation and air temperature values from these same GCMs over the 2018–2099 time period for each population, which we used to project population growth rate, after correcting future temperature to reflect discrepancies between GCM air temperature projections and soil temperature measured by iButtons (S1 Appendix; note that this correction assumes that the difference between soil temperature and climate projections is consistent across years). Because we were missing data for July soil temperature, we excluded July soil temperature from our annual and seasonal soil temperature calculations and used air temperature from SNAP data for hottest month temperature (which was often July; see S1 Appendix).

We synthesized the iButton temperature and SNAP data into ten climate variables describing 2015–2018 climate in each population: 1. average annual soil temperature; 2. average snow-free season soil temperature; 3. average snow-covered season soil temperature; 4. coldest month soil temperature; 5. warmest month air temperature; 6. total annual precipitation; 7. snow-free season precipitation; 8. snow-covered season precipitation; 9. coldest month precipitation; and 10. warmest month precipitation. Climate variables 1–4 were estimated from iButton data (and thus do not include July); 5–10 were estimated from SNAP data. We describe derivation of these climate variables in S1 Appendix.

We assessed the impact of these climate variables on *D*. *alaskana* vital rates using a model selection framework [19] in R [20]. For mean log size after one year of growth, variance in log size after one year of growth, probability of fruiting, and log number of fruits per size, we tested all possible subsets of a global model with log size in previous time step, average annual temperature, cumulative annual precipitation, and the interaction of average annual temperature and cumulative annual precipitation as predictor variables using the dredge function in the R package MuMIn [21], which allows comparison of multiple models using information theoretic approaches. We used AICc [22] to select a best-fit model. We repeated this model selection process for four other global models that included log size, temperature, and precipitation (and their interaction) during four time periods shorter than a year: 1) snow-covered season, 2) snow-free season, 3) coldest month, and 4) warmest month. We then selected, using AICc, the single best fit model across these five time periods, and used it in all subsequent analyses. Note that this approach does not allow for different windows of time to have effects on the same vital rate (e.g., warmest and coldest month temperatures cannot both affect a given vital rate), nor for quadratic temperature or precipitation effects reflecting an optimum, as we did not have sufficient data to test for these effects. We modified the model selection approach for probability of survival, which likely depends on both climate variables over the year of interest and, given the plant’s semelparity, the probability of fruiting in the prior year (which itself depends on the climate conditions in the prior year). Specifically, we tested the same 5 global models as described above, but also allowed these models to include terms for coldest month temperature and precipitation in the year prior, because coldest month conditions affected the probability of fruiting (see Results). The global model for survival also included log size in the prior year, the square of log size in the prior year, as well as interactions between log size and coldest month temperature, as well as between log size and precipitation in the year prior (S1 Appendix). We used a generalized linear model with a binomial distribution for probability of fruiting and survival, and a linear model for all other vital rates including variance in log size (note that using log size improved normality of residuals). Our approach assumes that variation in climate results in different vital rates across populations, rather than other non-climate factors that may also differ across populations, such as soil type. In addition, our approach assumes that populations respond similarly to climate, and does not allow for local adaptation resulting in different responses to climate.

We used the vital rate regressions to construct climate-driven population-specific integral projection models (IPMs) for current (2008–2022) and future (2086–2100) conditions using SNAP projections of climate over these time periods. While the demographic data span only one annual transition for each site, we used population growth rate over a longer time period (2008–2022) to represent current conditions, in an effort to guard against effects of unusual climate conditions in the year of the measurements. We substituted SNAP temperature values into the fitted regression equations to predict vital rates, correcting for population-specific differences between soil (iButton) and air (SNAP) temperature estimates, as described in S1 Appendix. Note that analogous to the vital rate fitting procedure, we allowed the warmest and coldest month to vary across populations, years, and GCMs. Because some vital rate predictions were unrealistic, we limited our vital rate values to 30% more or less than the observed maximum and minimum values for a size class in the discretized kernel (45% for binomial vital rates’ observed values). The degree of limitation does not affect our results (S1 Appendix). We calculated deterministic population growth rates for the current (2008–2022) and future (2086–2100) time periods using the mean kernel over these time periods. We included parameter uncertainty in the predictions by performing a parametric bootstrap, sampling 500 times from the multivariate Normal distribution of parameter estimates for each vital rate function and recalculating current and future population growth rates for each set of parameter estimates (note that this approach assumes that vital rates are independent). See S1 Appendix for more details on the construction of the IPMs.

We used an LTRE and a decomposition approach to estimate the relative contributions of each climate variable present in the vital rate functions to the change in growth rate for each population (where change is defined as future minus current). First, we used a LTRE approach to approximate the impact of each climate variable on population growth rate; specifically, we calculated the numerical derivative of the population growth rate with respect to each climate variable (using a perturbation approach; S1 Appendix) and multiplied it by the change in that variable (mean future, 2086–2100, minus mean current, 2008–2022, value). This approach assumes a linear response of population growth rate to climate. We then assessed the climate responsiveness as in Fig 1. Specifically, we regressed median annual population growth rate (deterministic lambda from the annual kernels; median calculated across the bootstrapped samples of regression coefficients) against splines of all annual climate variable present in the vital rate functions using a generalized additive model (GAM) in the mgcv package [23–25]. We used AIC to ensure that each population’s GAM was a better fit than an analogous linear model.

To account for the nonlinearity of climate responsiveness, we used a decomposition approach to estimate the relative contribution of each climate variable to the change in population growth rate for each population. We first calculated Δλ, the difference between population growth rate in 2086–2100 (future) and 2088–2022 (current), measured as deterministic λ of the mean kernel. We then calculated Δλ_c_, the contribution of climate variable c to Δλ, by comparing population growth rate of the current sequence to a ‘future’ population growth rate obtained by replacing one climate variable c with the 2086–2100 sequence, maintaining all other climate variables at the 2088–2022 sequence. Δλ_c_ isolates the impact of each climate variable in generating the difference between the future and current population growth rate, incorporating both climate responsiveness and differences in the magnitude of climate change across populations or climate variables. We repeated this decomposition for each population and each climate variable present in the vital rate functions. We found a high correlation (*R =* 0.85) between the sum of Δλ_c_ values across climate variables and Δλ, suggesting that the contributions are additive and the decomposition approach is reasonable.

We assessed the relative contribution of magnitude of climate change v. climate responsiveness in generating variation in climate change effects across populations using a multiple regression. For each climate variable, we ran a multiple regression using median Δλ_c_ as a response variable (where median is calculated across 500 bootstrap samples of regression coefficients). Predictor variables were the amount of change in a climate variable (Δc in Fig 1, mean future, 2086–2100, minus mean current, 2008–2022, climate conditions) and the climate responsiveness value for each climate variable (where climate responsiveness, Δλ/ Δc in Fig 1, is calculated using the GAM predictions, holding all non-focal climate variables at their mean). We used the sums of squares from an ANOVA analysis to partition the total variance of each Δλ_c_ into components due to degree of change in a climate variable, climate responsiveness, and their interaction. Note these results should be interpreted with caution, as the sample size is very low (5 replicate populations per multiple regression).

In the analyses in the main text, we use the average climate projections (across GCMs). Projections of population growth rates for individual GCMs are similar and shown in the S1 Appendix. All demographic data are archived on Figshare (10.6084/m9.figshare.13911755).

## Results

**Fig 3:**
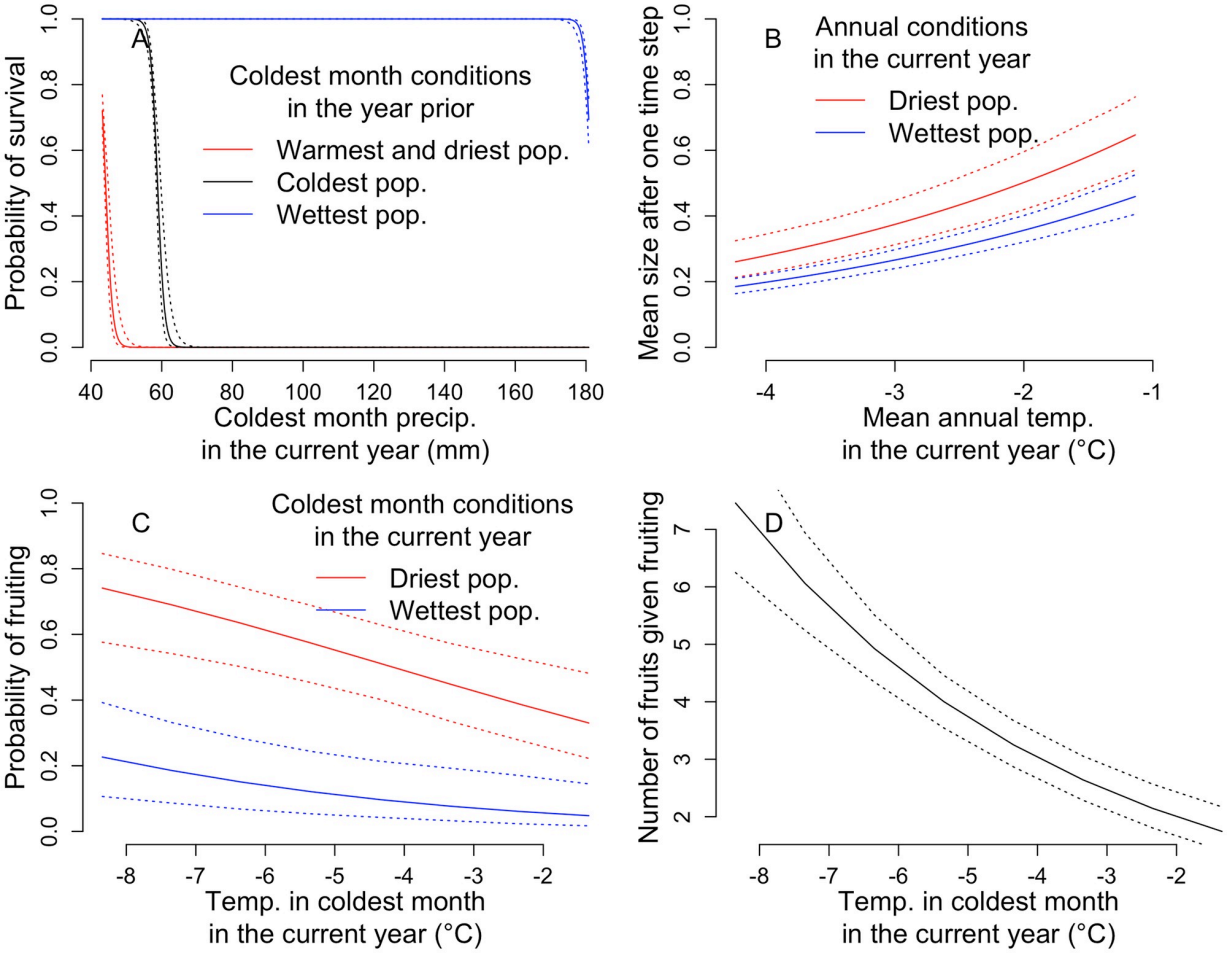
Effects of climate on vital rates. Vital rate relationships with climate for probability of survival (A), mean size after one year of growth (B), probability of fruiting (C), and number of fruits given fruiting (D) for a plant of median size (or for C, median size of fruiting plant). For A, we show how the impact of precipitation in the coldest month changes with coldest month conditions in the year prior. For prior year conditions, we use the observed temperature and precipitation for the coldest, wettest, and warmest/ driest populations (coldest: -9° C, 56.0 mm, wettest: -7° C, 158.2 mm, warmest/ driest: 1° C, 54.4 mm). For B and C, we show how the impact of temperature varies with the associated precipitation during the same interval, using values of precipitation from the driest or wettest populations: B driest: 662.8 mm, B wettest: 1431 mm, C driest: 43.2 mm, C wettest: 180.8 mm. See Table 1 for all parameter estimates, and S1 Fig in S1 Appendix for raw data.

We found effects of one or more climate variables on every vital rate that we tested for them, with the exception of variance in size after one year of growth (Table 1). Larger plants were more likely to fruit, but high temperature and high precipitation in the coldest month of the current year reduced the probability of fruiting (Table 1, Fig 3C). Larger size reduced the number of fruits per unit size given fruiting, and high temperature in the coldest month of the current year further reduced the number of fruits per unit size (Table 1, Fig 3D). Higher precipitation in the coldest month of the current year decreased survival (Fig 3A). Given that fruiting tended to be fatal, and that larger plants were more likely to fruit, the linear effect of size on survival was negative; a negative quadratic term suggests that survival may have been even lower for very large plants (Table 1). In addition, higher precipitation during the coldest month of the prior year increased current-year survival (Table 1, Fig 3A), which again can be explained by semelparity: higher precipitation in the prior year’s coldest month reduced the probability of fruiting in that year, which then increased survival in the current year. Counterintuitively, while high temperature during the previous year’s coldest month also reduced the probability of fruiting in the previous year (which should increase survival), this temperature variable actually decreased survival in the current year (Table 1, Fig 3A). In addition, survival exhibited positive interactions between size and both temperature and precipitation in the coldest month of the prior year (Table 1). Finally, mean size next year was smaller when annual precipitation was higher, but greater when annual temperature was higher (Table 1, Fig 3B).

**Table 1:** Vital rate functions.

Vital rate	Intercept	Log(size at previous census)	[Log(size at previous census)]^2	Precipitation in coldest month	Temperature in coldest month	Annual precipitation	Average annual temperature	Precipitation in coldest month (year prior)	Temperature in coldest month (year prior)	Precipitation in coldest month (year prior) x log (size in the previous time step)	Temperature in coldest month (year prior) x log (size in the previous time step)	Adjusted *R*^2^
Logit(survival)	-22.307	-0.697	-0.107	-1.022				1.262	-1.163	0.007	0.050	
	3.9534	0.116	0.022	0.176				0.217	0.205	0.001	0.015	
Mean log(size) at next census	1.188	0.727				–0.0004	0.293					0.73
	0.204	0.0202				0.0002	0.023					
Log(variance in log(size) at next census	–1.884	–0.118										0.006
	0.102	0.049										
Logit(fruiting)	–2.629	2.067		–0.017	–0.251							
	0.404	0.227		0.004	0.066							
Log(number of fruits per unit size given fruiting)	0.519	–0.218			–0.208							0.57
	0.166	0.072			0.023							
	Population											
	C	E1	E2	N	S							
Seedlings per fruit	0.193	0.333	0.160	0.097	0.196							

Coefficient estimates (first row) and their standard errors (second row) in the best-fit models for each vital rate. All coefficients are significantly different from zero at the 0.05 level. We also show estimates of seedlings per fruit (recruitment) for each population. Adjusted *R*^2^ of the best fit model is shown in the last column, if appropriate.

**Fig 4:**
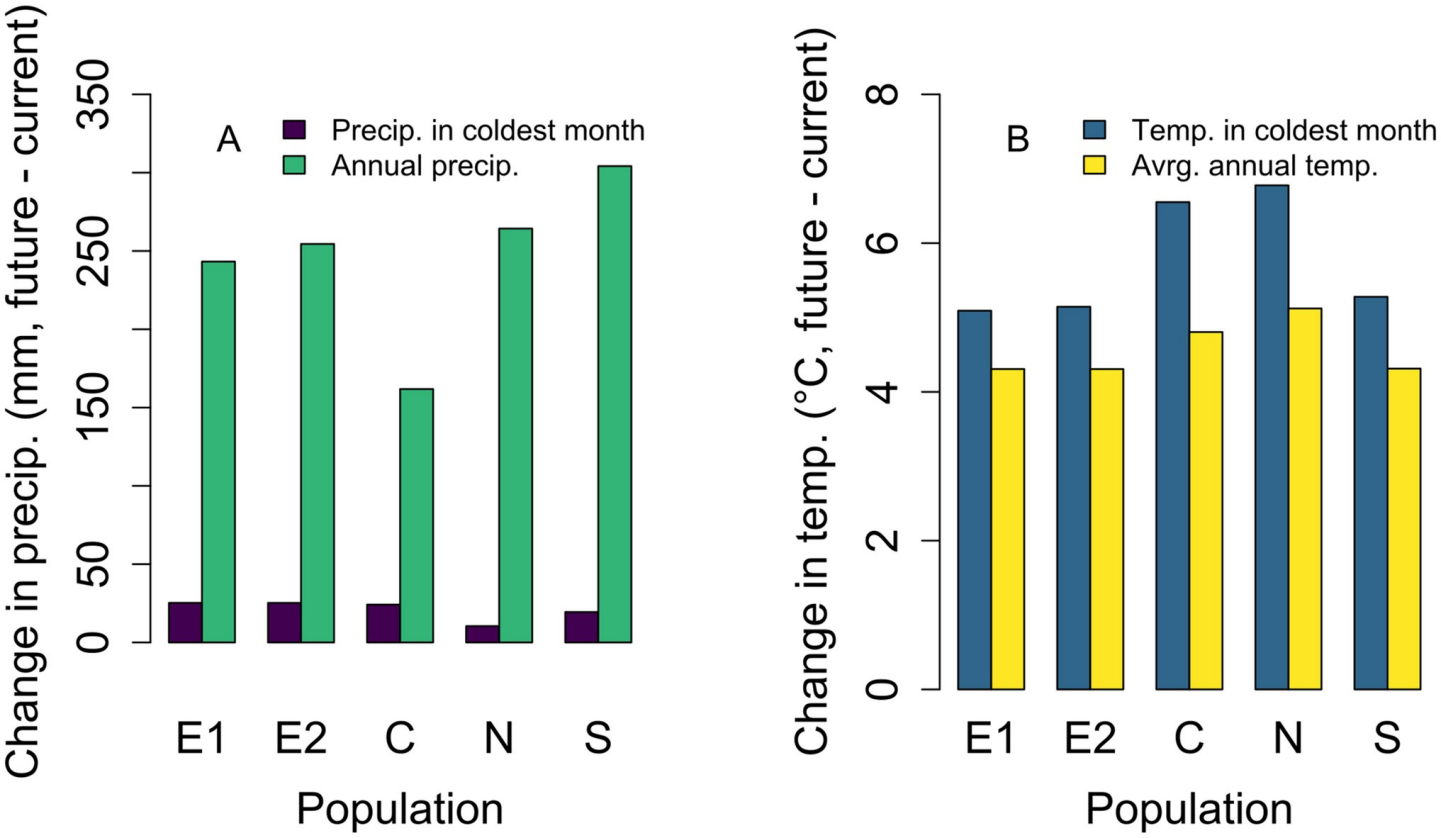
Rate of climate change. Change (mean future, 2086–2100, minus mean current, 2008–2022) in precipitation (A) and temperature (B) conditions for five populations for each climate driver present in the vital rate functions. Populations are arranged along the x-axis by increasing current average annual temperature; for example, the E1 population has the coldest average annual temperature over the current period, and the S population has the warmest average annual temperature over this period. See S1 Table in S1 Appendix for raw data.

**Fig 5:**
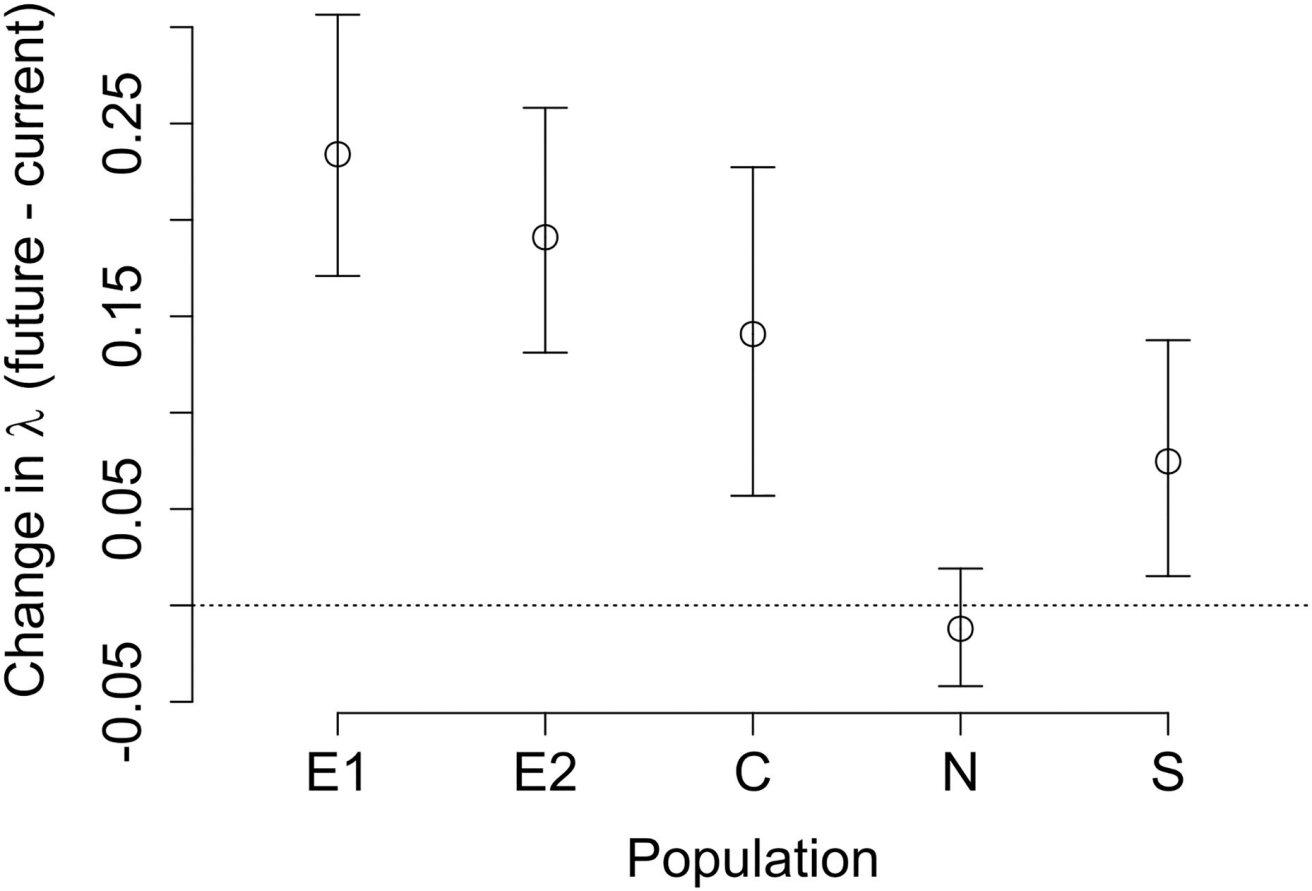
Change in population growth rate due to climate change. Impact of climate change on population growth rate for all populations, expressed as the difference between future (2086–2100) and current (2008–2022) population growth rates. Points indicate the mean difference and error bars indicate 95% confidence intervals of differences (calculated across 500 bootstrapped samples from the distributions of model coefficients). As in other figures, populations are arranged by increasing current average annual temperature. See S2 Fig in S1 Appendix for population growth rates, and S3 Fig in S1 Appendix for GCM-specific results.

Climate is predicted to be warmer and wetter in all populations on average, but the magnitude of temperature increases and the timing of changes in precipitation differ among populations (Fig 4). Mean annual temperature increase is projected to be higher in all populations by the end of the current century, ranging from 4.3 to 5.13° C. Increases in coldest month temperature are even more dramatic, ranging from 5.1 to 6.8° C. The N population will likely experience the most substantial increases in both annual and coldest month temperature over the 2018–2099 period, while the S, E1, and E2 populations will only experience moderate increases. While annual precipitation is predicted to increase in all populations, the magnitude and timing of precipitation changes will differ across populations (Fig 4). For example, annual precipitation will increase the least in the C population, though changes in coldest month precipitation are still high (Fig 4). In spite of these differences in climate change magnitude across populations and in climate effects on different vital rates, we project significant increases in population growth rate in a future climate at all populations but the N population, with particularly large increases at the E1 and E2 populations (Fig 5).

**Fig 6:**
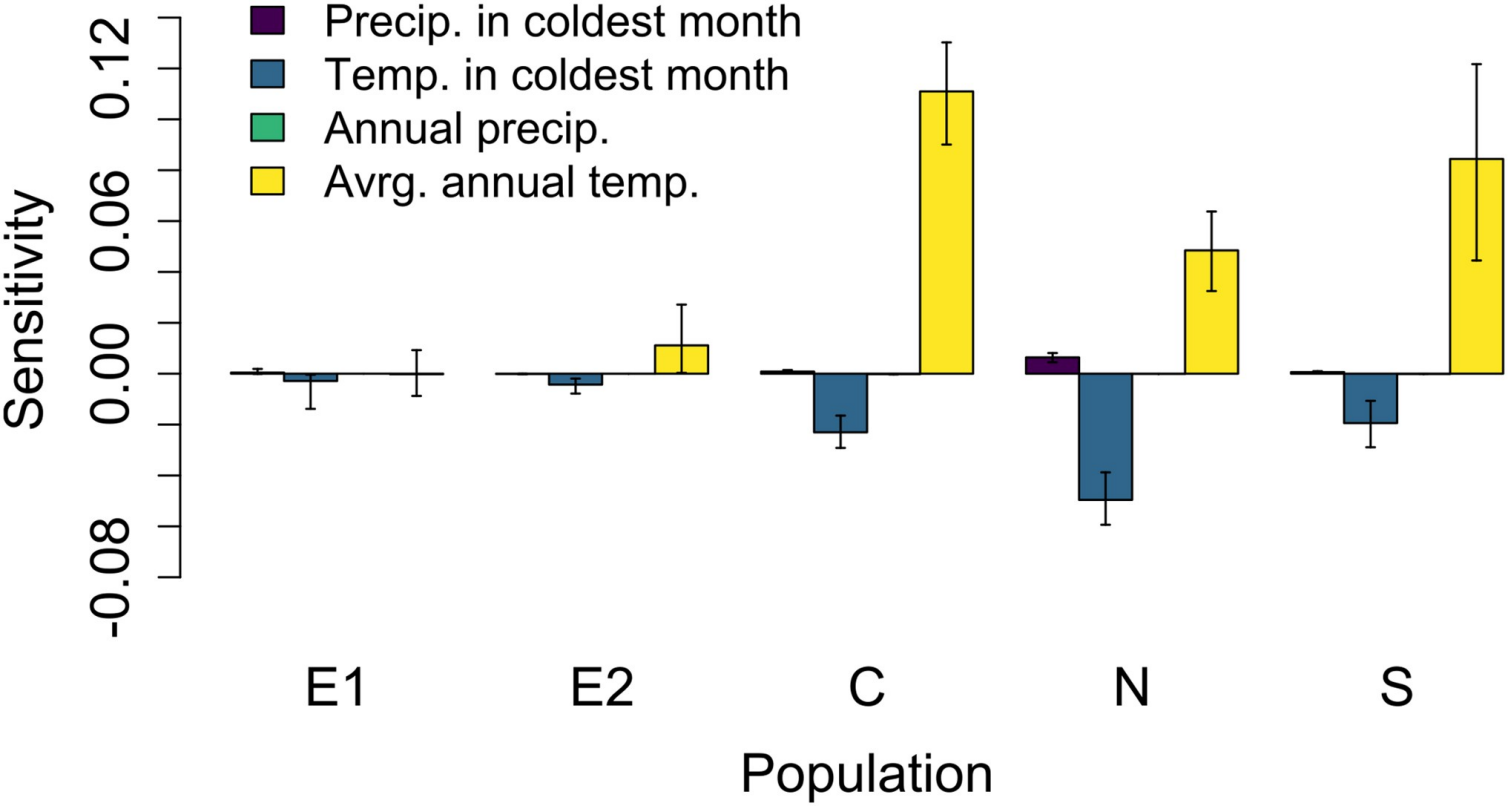
Sensitivity to climate variables at current (2008–2022) climate conditions. Sensitivities were calculated using a perturbation approach using kernels from the current time period. We show the mean sensitivity across 500 bootstrap replicates; bars indicate the 95% confidence intervals on sensitivities. As in other figures, populations are arranged by increasing current average annual temperature.

Sensitivities to climate variables differed dramatically in magnitude and sign across populations. Sensitivity to average annual temperature was highest at the C, N, and S populations, particularly at the C and S populations (Fig 6). By contrast, sensitivity to average annual temperature was very low (or even negative) at the E1 and E2 populations (Fig 6). Sensitivities to other climate variables were low, but also differed across populations in magnitude and sign (Fig 6).

**Fig 7:**
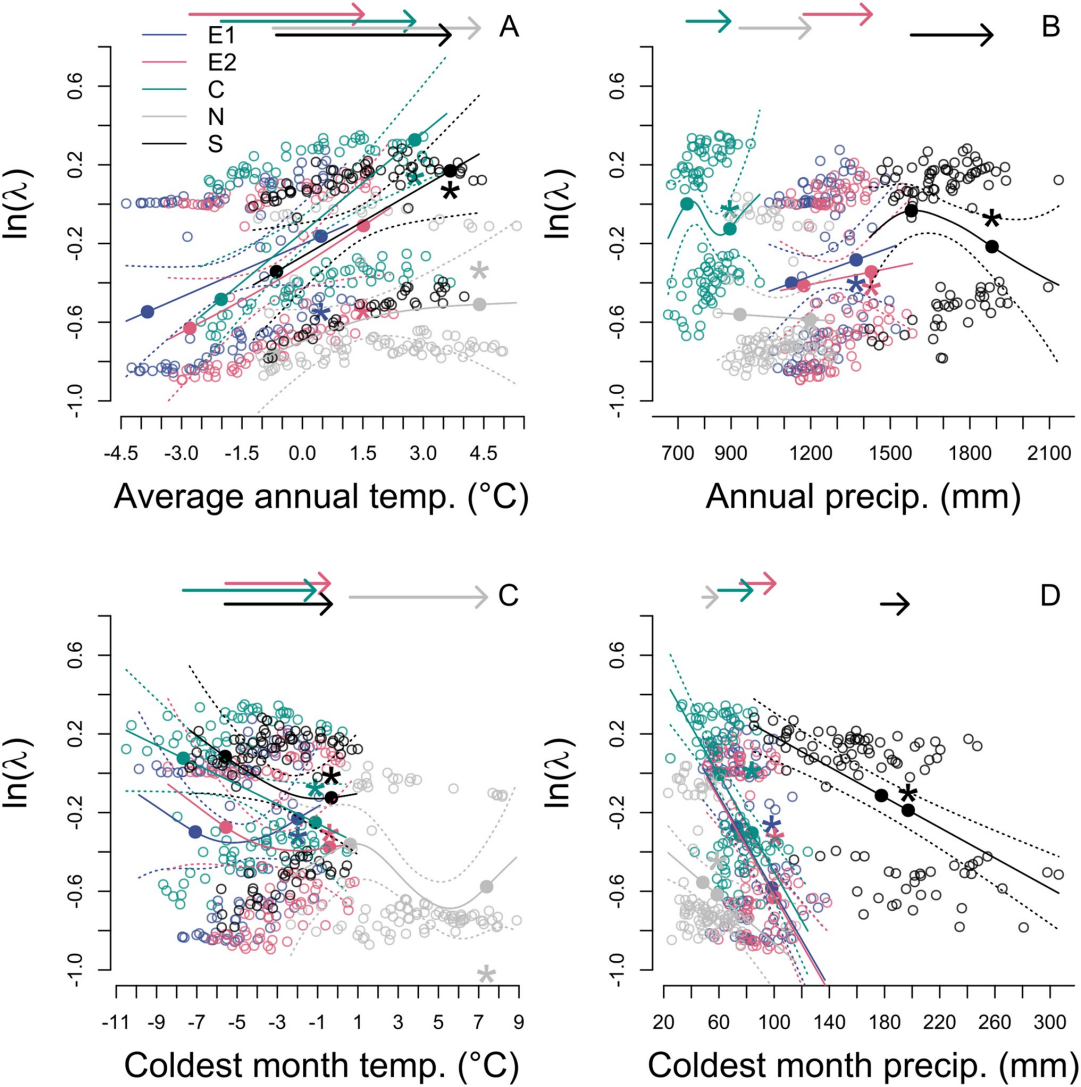
Nonlinear responses of *D*. *alaskana* to climate. Response of annual population growth rates (λ) to changes in aspects of temperature and precipitation that affect vital rates. Unfilled points represent medians (across bootstrapped regression coefficients) of the annual population growth rates vs. the current and future GCM climate values for each year from 2008 to 2099; curves represent predictions of population-specific GAMs with non-focal climate variables held at their mean values. Arrows on the top of the figures represent the magnitude of climate change at each population; start of the arrow is at the mean climate condition for the current period (2008–2022) and end of the arrow is at the mean climate condition for the future period (2086–2100). Filled points represent population growth rates predicted by the GAM at current and future mean climates, with an asterisk indicating future population growth rate predicted by an LTRE approach.

We found nonlinearity in the responses of population growth rate to climate, which often resulted in a poor performance of LTRE approximations. Our comparisons of GAM and linear models showed support for nonlinearity in climate responses. Note that results should be interpreted with caution, as including coldest month conditions in GAMs did not satisfy some assumptions, but GAMs with only annual conditions satisfied all assumptions and showed similar results (S1 Appendix). Some responses of population growth rate to climate were roughly linear. For example, responses to average annual temperature at all populations besides the N population (Fig 7A) were roughly linear. However, many other relationships were highly nonlinear, showing unimodal responses (effect of coldest month temperature at the S and E2 populations, Fig 7C), decelerating responses (average annual temperature at the N population, Fig 7A), or more complex relationships (annual precipitation at the C population, Fig 7B). For most climate variables and populations, the LTRE approximation was inaccurate (Fig 7), resulting in a relatively weak correlation between the LTRE-predicted change in population growth rate and the GAM-predicted change in population growth rate (Pearson’s *R* = 0.62, n = 2500, S4 Fig in S1 Appendix). The direction and magnitude of bias of LTRE approximations differed dramatically across populations and across climate variables (Fig 7).

**Fig 8:**
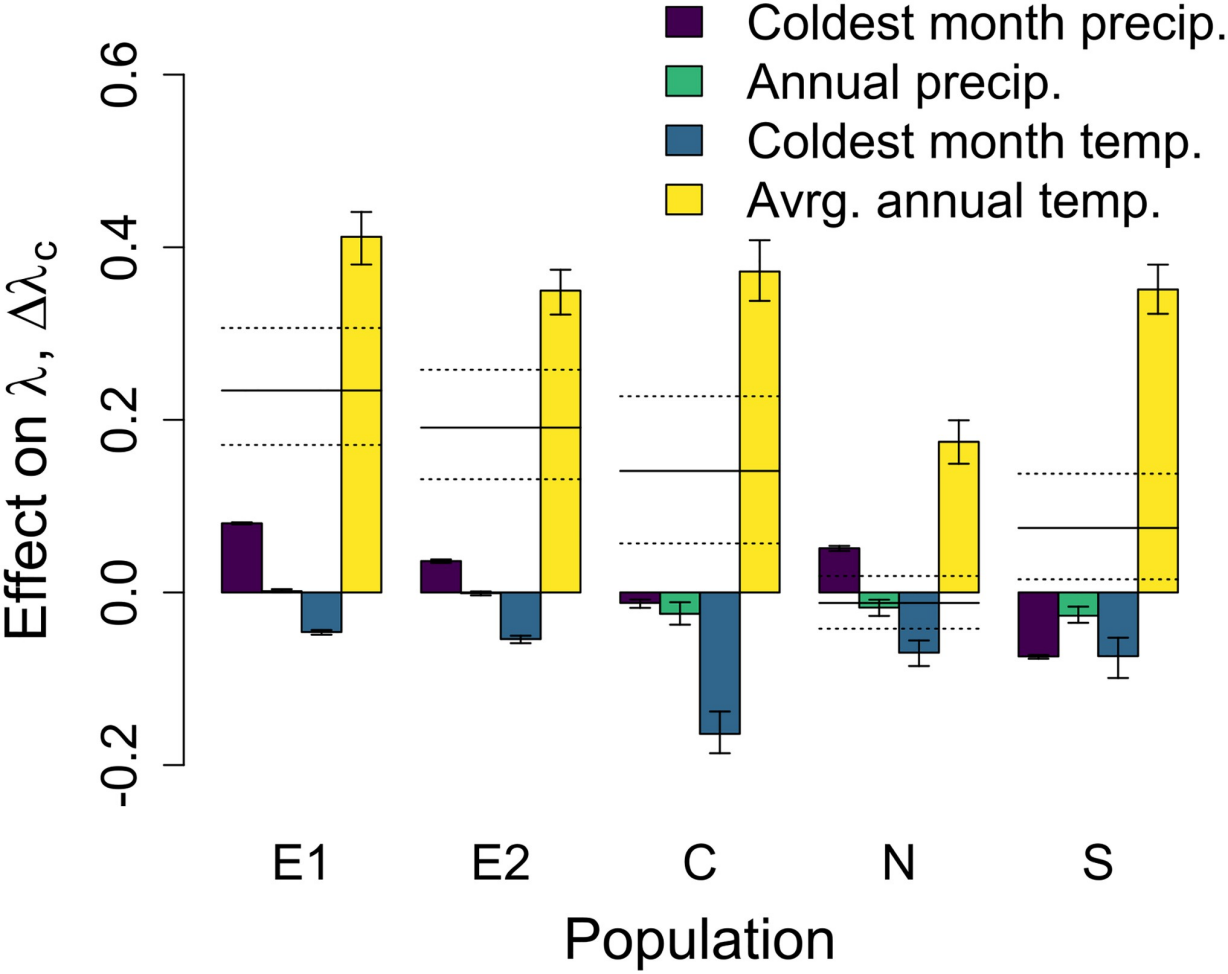
Δλ_c_, the effect of each climate variable on the change in population growth rate. We show Δλ_c_, calculated for each climate variable changing current (2008–2022) climate variables to future (2084–2099) climate variables, one climate variable at a time. Bars indicate the mean Δλ_c_ and error bars indicate the 95% confidence intervals of Δλ_c_s, calculated across bootstrapped parameter estimates. Horizontal lines indicate the mean Δλ, future- current population growth rate, and dotted lines indicate the 95% confidence intervals of Δλ, calculated across bootstrapped parameter estimates. In both panels, populations are arranged in the same order as in prior figures (increasing current average annual temperature).

Differences in climate conditions across populations resulted in variation in the response of population growth rate to particular climate conditions, and the resultant variation in climate responsiveness modulated the impact of each aspect of climate on population growth rate. Across populations’ climate conditions, the relationship between population growth rate and a particular climate variable differed in magnitude and in degree of nonlinearity. For example, population growth rate increases linearly with changes in average annual temperature at most populations, but the N population’s response is decelerating (Fig 7A), resulting in only a moderate effect of average annual temperature on population growth rate (Fig 8). Supporting these findings, the direction and degree of bias of LTRE approximations differed across populations’ climate conditions (S4 Fig in S1 Appendix), depending on whether the relationship between climate and population growth rate was linear, concave, or convex (Fig 7). For example, in Fig 7B, the S and C populations’ response to annual precipitation is highly nonlinear; these populations’ LTRE approximation predicted higher future population growth rate than is likely to occur. By contrast, the E1 and E2 populations’ response is quite linear; these populations’ LTRE approximation predicted lower future population growth rate than is likely to occur.

Average annual temperature was the most important driver of changes in population growth rate, with weaker effects of other climate variables. Average annual temperature strongly increased population growth rate at all populations, though the effect size, Δλ_c_, varied (Fig 8). In fact, increases in average annual temperature are predicted to increase population growth rate so substantially that they are sufficient to counter the negative effects of changes in all other climate variables at all populations besides the N population. Coldest month temperature was the most important climate variable that reduced population growth rate, though again Δλ_c_ varied across populations (Fig 8).

Among-population variation in climate responsiveness to average annual temperature is predicted to drive differences in climate change effects across populations. The multiple regression indicated that for average annual temperatures, climate responsiveness, rather than change in climate conditions, will be the key determinant of change in population growth rate (Table 2). For example, at the N population, climate responsiveness to average annual temperatures is low (Fig 7A). Low climate responsiveness leads to a small Δλ_c_ at the N population (Fig 8), resulting in weaker overall effects of climate change at this population (Δλ is low, Fig 5). This effect occurs even though the N population is projected to experience the greatest increase in average annual temperature (Fig 4B). For other climate variables, both the degree of change in climate conditions and climate responsiveness are key determinants of changes in population growth rate (Table 2).

**Table 2:** Role of climate responsiveness v. rate of climate change.

Climate variable	Climate responsiveness	Change in climate conditions	Change in climate conditions x climate responsiveness	Residual
Average annual temp. (°C)	**0.84933**	0.08188	0.06631	0.00248
Annual precip. (mm)	0.20552	**0.39518**	0.0799	0.3194
Coldest month temp. (°C)	**0.60387**	0.03245	0.28828	0.0754
Coldest month precip. (mm)	0.12438	**0.50025**	0.22179	0.15358

Proportion of variance in the contribution of climate variables to change in population growth rate (Δλ_c_) attributable to climate responsiveness v. the change in climate conditions (Δc, mean future, 2086–2100, minus mean current, 2008–2022, conditions), and the interaction between the two. Climate responsiveness is obtained by regressing median annual growth rates (median is across 500 bootstrapped sets of parameter estimates) on annual climate conditions using a GAM (Fig 7), and dividing Δλ by Δc, as in Fig 1. For all multiple regressions, the response variable, Δλ_c_ (contributions of climate variables to Δλ) is the median across 500 bootstrapped sets of parameter estimates. Bold indicates which effect has the highest proportion of variance for each climate variable.

## Discussion

Temperature and precipitation have a mix of positive and negative effects on *D*. *alaskana* vital rates, consistent with a variety of other studies. Our findings of positive temperature effects on survival and growth, but negative temperature effects on reproduction, are similar to results found in other alpine plants. Doak and Morris [26] found that higher temperature decreases reproduction in the wide-ranging alpine plants *Silene acaulis* and *Polygonum viviparum*, but increases growth rates, perhaps due to longer and warmer growing seasons. In *D*. *alaskana*, we also see a lagged negative effect of coldest month temperature on survival, which is surprising given that coldest month temperature reduces the probability of fruiting, which should in turn increase survival given the plant’s semelparity. Precipitation also impacts vital rates, with a mix of positive and negative effects. Positive effects of precipitation could reflect increased water availability or protection from damaging frosts due to high snowpack [27–29]. Alternatively, a larger snowpack could lead to a shorter growing season, resulting in negative effects of precipitation. In *D*. *alaskana*, we also see a strong lagged positive effect of coldest month precipitation on survival, which likely arises because precipitation in the coldest month reduces the probability of fruiting, which then increases survival the subsequent year. Consistent with our findings, other work also shows a mix of positive and negative effects of precipitation on different vital rates [30]. Surprisingly, we see no evidence for precipitation x temperature interactions; other studies have suggested that such interactions might be critical in alpine species. For example, increasing temperature could accelerate melt-out and negate any negative impact of precipitation [31].

In spite of the mix of positive and negative effects of temperature and precipitation, population growth rate across the species’ range is projected to be higher under future climate conditions (higher temperature and precipitation). These positive effects appear largely mediated by increases in average annual temperature (Fig 8), likely resulting from the positive effect of average annual temperature on mean growth rate (Table 1). Consistent with our findings, a recent review showed that many high- and mid-latitude populations across many taxa have responded positively to increased temperature under current climate conditions. In particular, plants responded more positively to temperature increases than any other taxa studied (invertebrates, fishes, amphibians, birds, or mammals; [32]). Our results further suggest that future climate change will reduce *D*. *alaskana*’s extinction risk, an unexpected result considering that uncommon species such as *D*. *alaskana* are more likely to go extinct than common species [33]. However, both current and future population growth rates were below unity for most populations (other than future population growth rate at the S and C populations, S2 Fig in S1 Appendix), suggesting that even with increases in population growth rate with climate change, most *D*. *alaskana* populations, besides those in the most favorable conditions, will decline to extinction. While current population growth rates less than one are somewhat counterintuitive, it is possible that suboptimal historical conditions (climate or otherwise) have resulted in current low population growth rates. This prediction is consistent with the likely extirpation of at least one population from previously occupied habitat (A. Louthan, pers. obs.). Alternatively, lower-than usual growth rates during the years of our study, seed bank dynamics, or unaccounted for immigration could result in low estimates of current population growth rates.

Variation in the impacts of climate change on population growth rate is driven primarily by differences in populations’ climate responsiveness, suggesting that future studies should recognize that climate responsiveness, and thus the degree to which current response to climate predicts future response, could vary across populations. Differences in climate responsiveness across populations are not surprising; we know that interspecific variation in life history characteristics can modulate the impact of a given amount of climate change. For example, species that are shorter lived [34] or have shorter generation times [35] tend to be more impacted by changes in environmental conditions, such as climate change. We also know that at the intraspecific level, life history characteristics can vary substantially across populations [12], meaning that populations could vary in their responsiveness to changes in environmental conditions. For example, perhaps populations with lower survival (and thus shorter generation times) have higher climate responsiveness. Among-population variation in climate itself can generate variation in life history that then modulates the impact of climate. For example, relative to other populations, the N population is less sensitive to the positive effects of average annual temperature on population growth rate (Fig 8), likely because changes in other climate variables will modulate its sensitivity to mean annual temperature. Namely, large increases in coldest month temperature at this population (Fig 4B) will lead to reduced probability of reproduction of those larger plants (Table 1), tempering the positive effects of higher average annual temperature on reproduction (mediated through larger sizes due to higher mean growth rates; Table 1).

Nonlinear responses of population growth rate to climate are very common, and the degree of nonlinearity, as well as the shape of the nonlinear relationship, varies dramatically across populations. The sensitivities to climate variables at current climate conditions are very different, in both magnitude and sign, than the climate responsiveness, which incorporates the entire range of temperature and precipitation conditions. For example, at the E1 population, the (linear) sensitivity to average annual temperature at current climate conditions is negative (Fig 6), which suggests that increases in average annual temperature should decrease population growth rate. However, when we incorporate nonlinearities in the response to changes in temperature, we see a net positive effect of increases in average annual temperature on population growth rate (Fig 8). The direction and magnitude of bias in LTRE predictions varies across populations (due to variation in their climate conditions; Fig 7A), with similar effects for other climate variables (Fig 7B–7D). While the possibility of nonlinearities in responses of population growth rate to climate and other drivers are often acknowledged in population-level analyses, we know little about their prevalence [36, 37], let alone the degree to which the presence or degree of nonlinearity in responses differs across populations. Particularly concerning is the growing body of evidence that nonlinear population responses to climate are common and widespread [26, 38], and that it is difficult to quantify how large increases in temperature will affect population dynamics due to the dearth of data on population responses at historically unprecedented temperatures [39]. While past work has shown that a linear approximation is generally reasonable when calculating elasticities to vital rates [40], these analyses ignore covariances among vital rates, which are present in *D*. *alaskana*, as well as any other species in which one climate variable affects multiple vital rates (as likely occurs for most species). Thus, our results suggest that population-specific nonlinearities (effected in our study by variation in climate conditions) may be critical in mediating overall population response to changes in climate or other drivers for a variety of species.

Our metrics of climate responsiveness and of sensitivity to climate variables assume that variation in climate response arises only from climate differences across populations, but we know that natural selection could also lead to variation in response across populations. Only a few studies quantify how sensitivity varies across populations across a species’ range (e.g., ref. [41]), and fewer still quantify whether this variation arises from local adaptation or simply variation in driver values across a species’ range. Past work suggests that populations should be relatively insensitive to climate drivers that were historically highly variable [42]. We see some support for this pattern, with the N population (which has likely experienced the highest historical climate variability, due to its inland, high-latitude location) less responsive to some climate drivers than other populations (the S and C populations, which are coastal, were likely historically buffered from climate variability due to proximity to the ocean). Similarly, in Chinook salmon, juvenile survival in different populations depends on completely different climate drivers, which results in divergent responses to climate changes [43]. Whether differential responses of salmon juvenile survival to climate arise from local adaptation or not is unclear. Regardless, the salmon study shows that variation in climate change effects on population growth rate can arise from differences among populations in the slope of the vital rate function v. climate relationships, rather than only by variation in climate response caused by among-population variation in mean climate conditions, as occurs in *D*. *alaskana*. In addition to local adaptation, other among-site differences in non-climate drivers, such as soil or elevation, are confounded with climate differences in our study, because we only have one year of demographic data per site.

Spatial variation in some climate variables’ impacts was due to variation in climate change magnitude across space (Table 2). Other studies have found that rates of warming vary across space, with high-latitude, inland, or wind-protected locations experiencing higher rates of warming [4]. Additionally, rates of population declines in birds and mammals are greater in locations where temperature has increased more [5]. Our work also suggests that differential rates of warming across seasons could have implications for climate effects on population growth rate. We found that increases in average annual temperature increase future population growth rate, but increases in coldest month temperature actually decrease population growth rate (Fig 8). Consistent with this finding, other studies have shown that seasonality of climate changes, relative to the life history of the organism, matters; for example, egg viability of two butterfly species decreased with extreme high temperature, but high mean temperature had positive effects on larval growth rates [44]. Similar effects have been observed for phenological responses [45] and for crop yield [46].

Our work has important implications for predicting effects of climate change on populations across a species’ range. Effects of climate change on *D*. *alaskana* are universally positive and nonlinear, mediated primarily by spatial variation in climate responsiveness. Variation in the rate of climate change also contributes to differential impacts of climate change across populations. Thus, our work suggests that current responses to climate may not continue indefinitely under continued changes in climate, but that populations may differ dramatically in their responses to future climate, even if their current responses to climate are very similar to one another. This is important because often conservationists only have data on current responses to climate, rather than future response to climate. All of these factors are likely to generate idiosyncratic responses to climate across populations. Thus, our work may provide a framework for understanding why many species do not show predictable shifts in their geographic range with climate change [2, 47, 48].

## Supporting information

S1 Appendix (DOCX)
