# Supplementary material for: Climate change impacts on population growth across a species’ range differ due to nonlinear responses of populations to climate and variation in rates of climate change
Source: PLoS One. 2021 Mar 3;16(3):e0247290. doi: 10.1371/journal.pone.0247290 (PMC7928526; doi:10.1371/journal.pone.0247290)
Supplement: S1 Appendix — (DOCX) [file pone.0247290.s001.docx]

*Size metrics & vital rate fitting*

For our global model for survival, we included the square of log size in the prior year because we suspected mortality following fruiting might be more common for smaller plants. We did not have enough data to fit interactions between a squared size term and coldest month temperature and precipitation in the year prior.

We were concerned that our measurements of rosette height were prone to measurement error, as it was difficult to measure height consistently in rocky terrain. We used a model selection approach to test whether the size metric used in the main analyses (estimated volume, defined as height x basal area of a rosette, pi*radius of rosette^2, summed across all rosettes) was a better predictor of vital rates than an alternative size metric (estimated basal area, summed across all rosettes). For all vital rates besides growth, we found that our estimate of volume enjoyed more support than our estimate of basal area. Namely, for each of these vital rates, we tested whether the given response variable was better predicted by volume or by basal area, using the same structure of models as for the vital rate analyses (with the exception of log fruits per size; for this response variable, we tested whether log fruits was best predicted by volume in the previous time step v. basal area in the previous time step). Note that the structure of models for the survival response included a linear and a quadratic effect of size. Volume, the size metric used in the main analysis, was always a better fit. We did not test whether mean or variance in growth was better predicted by volume, because the response variables (change in size or residuals of change in size) depend on the size metric itself and thus the models were not directly comparable using AICc.

*Details on climate data*

We obtained estimates of monthly air temperature and precipitation for the Business-As-Usual Emissions Scenario (RCP 8.5) from the CMIP5/AR5 models (Coupled Model Intercomparison Project Phase 5/ Assessment Report 5) from the University of Alaska’s Scenarios Network for Alaska and Arctic Planning group (SNAP 2019). SNAP climate projections were downscaled using CRU CL v. 2.1 climatological datasets from 1961-1990 as the baseline, generating 2km x 2km spatial resolution climate projections from 2006-2099.

We corrected the SNAP data to better represent soil temperature data using population specific differences between SNAP and iButton temperatures. The SNAP temperature data could differ from the iButton temperature data due to differences between soil and air temperatures, but also due to elevation of the population, insolation, or other microsite characteristics. We only had temperature data from both iButton and SNAP sources for the 2016-2017 or 2017-2018 period, depending on population. For each population, climate variable, and GCM (including the mean GCM), we calculated a difference between the iButton and SNAP data, and then used this difference to ‘correct’ all SNAP data used in population projections. Note that we did not measure precipitation or soil moisture at any population, so all precipitation data used in projections was obtained from SNAP.

We then used these metrics to fit vital rate functions using the 2016-2018 data. All SNAP-derived climate variables are averaged across GCMs to obtain a 2016-2017 or 2017-2018 value. In addition, we were missing iButton data from some populations for July, so hottest month temperatures were always air temperatures derived from the SNAP data. We identified the coldest and warmest month for each population from the 2016-2017 or 2017-2018 SNAP data (averaging temperatures across the 5 GCMs). In order to account for any bias generated by the regressions of climate on snowmelt and snowfall timing, we determined the snow-free and snow-covered months using the MODIS regression described in the next paragraph.

To estimate timing of snowmelt and snowfall for a given year, we regressed dates of snowmelt and snowfall over the 2007-2018 period (obtained from satellite images of daily snow cover) on temperature and precipitation during the year (obtained from SNAP climate projections). We estimated dates of Spring snowmelt and Fall snowfall using satellite images of daily snow cover for 2007-2018 at each of these populations from the Daily Snow Cover Moderate Resolution Imaging Spectroradiometer Dataset (MODIS; Hall and Riggs 2016). We used the across-GCM mean of the SNAP projections over the 2007-2018 period to determine the relationship between timing of snowmelt and snowfall v. climate using a model selection approach. For a global model for month of snowfall, we regressed 2007-2018 month of snowfall (from MODIS) on cumulative September-December precipitation, mean September-December monthly temperature, and their interaction (from SNAP). For a global model for month of snowmelt, we regressed 2007-2018 month of snowmelt on cumulative September-May precipitation, mean September-May temperature, and their interaction. We tested all possible subsets of the global models against one another, selecting a best-fit model using AICc for both month of snowfall and snowmelt. We then used this best-fit model to predict snowmelt dates and snowfall dates for all years and populations in our study, including both the vital rate fits and the IPM projections. Similar to the vital rate fits described in the main text, each year was defined by the August 1 to July 31 interval; for example, we regressed snowfall month in 2017 and snowmelt month in 2018 against temperature and precipitation from August 1 2017- July 31 2018. The snowfree season included the month during which snow melted, but not the month in which snow fell. Snowmelt and snowfall timing were consistent across years (snowmelt was always May or June, snowfall always September or October).

*Details on IPM construction and linear sensitivity analysis*

Our kernels used log size as a size variable and had an additional class for seedlings. We used the population-specific estimates of seedlings per fruit in the year prior as an estimate of recruitment (using the across-population average for the S population, for which we were missing recruitment data). Seedlings survived at the average rate across populations, and survivors joined the size distribution with a mean and variance in size equivalent to the mean and variance across all populations in the field study. Because some of the fitted vital rate functions predicted unrealistic values for novel climate conditions, we limited all vital rates to 30% above or below their observed maxima or minima, respectively (for binomial vital rates, 45% above or below the predicted probability for that size class). The primary conclusion of our paper (current sensitivities to climate are a poor approximation for future population growth rate; namely, correlation between LTRE-predicted growth rate and GAM -predicted growth rate is low, *R*<0.62) is robust to changing the 30%/45% bounds to be more restrictive (25%/ 37.5%) or less restrictive (35%/52.5%). Our kernel had 40 mesh points, with upper and lower bounds at the log of 10% greater than the maximum and 10% less than the minimum sizes observed during the study, respectively. We renormalized predicted size in the next time step to avoid eviction (Williams, Miller, & Ellner, 2012). To prevent complete eviction below the lower size bound (where all predicted sizes, not just mean size after one year of growth, were below the lower bound of the kernel), we assumed in these cases that all individuals transitioned to the smallest size class (Williams et al., 2012). No complete eviction over the upper limit occurred. Formally, our IPM kernel can be represented by the equation: K(z′,z) = s(z)* G(z′,z)+p(z)*b(z)*r*C(z′), where z is size in the first time step, z’ is size in the next time step, s(z) is size-dependent survival, G(z’,z) represents growth from size z to size z’, p(z) is the size-specific probability of reproduction, b(z) is the number of fruits given fruiting, r is seedlings per fruit, and C(z’) is the size distribution of seedlings.

We estimated linear sensitivities of current (2008-2022) population growth rate to climate using a perturbation approach. Namely, we perturbed each climate variable one at a time, by either + or - 5% of the 2008-2022 mean climate value, for each year of the 2008-2022 simulation, and quantified the change in population growth rate. We calculated sensitivity as the change in population growth rate divided by the actual change in the climate variable, averaged over the two perturbations (up and down). Following an LTRE approach, we assumed that each climate variables’ impact on population growth rate could be approximated by multiplying the sensitivity to that climate variable (under current conditions) by the change (future- current) in that climate variable. Consistent with our findings of nonlinear responses to climate, the product of sensitivity to a climate variable and change in that climate variable, summed across all climate variables, did not correlate well with the impact of climate change (*R*= 0.012).

*Alternate GAM formulation*

Because our GAM fits that used all four climate variables as (smoothed) predictor variables suffered from some problems with the residuals (namely, a slight linear trend in the residuals v. predictor value), we conducted another suite of analyses using GAMs fit to only (smoothed) annual predictor variables, which satisfied all assumptions. Note that for most of these modified GAMs, 4/5, a linear fit was a better approximation than the nonlinear fit, according to AIC, but the nonlinear fit was always within 0.91 AIC units of the linear fit. We show the fits of these functions in S5 Fig. We show the LTRE approximation predictions, but caution that these fits, which take into account concurrent variation in multiple climate variables, are not analogous to the LTRE predictions, which isolate effects of one climate variable at a time.

Citations:

Hall, D., Riggs, G. (2016). MODIS/Terra Snow Cover Daily L3 Global 500m Grid, Version 6. Boulder, Colorado USA. NASA National Snow and Ice Data Center Distributed Active Archive Center. doi: 10.5067/MODIS/MOD10A1.006. Accessed August 4, 2019.

Scenarios Network for Alaska and Arctic Planning, University of Alaska. ***2019. Projected Monthly Temperature Products- 10 min CMIP5/AR5.*** Retrieved ***5/2/2019.*** from <http://ckan.snap.uaf.edu/dataset/projected-monthly-temperature-products-10-min-cmip5-ar5>

Williams, J. L., Miller, T. E. X., & Ellner, S. P. (2012). Avoiding unintentional eviction from integral projection models. *Ecology*, *93*(9), 2008–2014. doi: 10.1890/11-2147.1

**Table S1.** Current, future, change, and percent change in climate conditions at each population for each of the four climate variables used in vital rate functions. Percent change is measured as 100* (future-current)/ current values, where temperature is measured in Kelvin.

|  | Current | | | | future | | | | Change (future – current) | | | | % Change (future – current) | | | | |
| --- | --- | --- | --- | --- | --- | --- | --- | --- | --- | --- | --- | --- | --- | --- | --- | --- | --- |
| population | temperature in coldest month (°C) | precipitation in coldest month (mm) | annual precipitation (mm) | Mean annual temperature (°C) | temperature in coldest month (°C) | precipitation in coldest month (mm) | annual precipitation (mm) | Mean annual temperature (°C) | temperature in coldest month (°C) | precipitation in coldest month (mm) | annual precipitation (mm) | Mean annual temperature (°C) | temperature in coldest month (K) | precipitation in coldest month (mm) | annual precipitation (mm) | Mean annual temperature (K) |  |
| C | -7.67 | 59.73 | 733.69 | -2.02 | -1.12 | 83.9 | 895.51 | 2.79 | 6.55 | 24.17 | 161.82 | 4.81 | 2.467 | 7.261 | 16.072 | 1.774 |  |
| S | -5.59 | 177.74 | 1579.14 | -0.64 | -0.31 | 197.14 | 1883.34 | 3.67 | 5.28 | 19.4 | 304.2 | 4.31 | 1.973 | 4.303 | 16.423 | 1.582 |  |
| N | 0.62 | 48.31 | 933.21 | -0.73 | 7.39 | 58.74 | 1197.51 | 4.4 | 6.77 | 10.43 | 264.3 | 5.13 | 2.473 | 3.245 | 21.909 | 1.883 |  |
| E1 | -7.08 | 72.97 | 1127.99 | -3.84 | -1.98 | 98.26 | 1371.2 | 0.46 | 5.1 | 25.29 | 243.21 | 4.3 | 1.917 | 7.307 | 17.358 | 1.597 |  |
| E2 | -5.55 | 75.39 | 1174.33 | -2.79 | -0.41 | 100.64 | 1428.8 | 1.51 | 5.14 | 25.25 | 254.47 | 4.3 | 1.921 | 7.245 | 17.58 | 1.59 |  |

**Table S2.** Correlations among climate variables for each population, using data from all years.

| C pop. | annual precip. | coldest month temp. | coldest month precip. |
| --- | --- | --- | --- |
| annual temp. | 0.813 | 0.899 | 0.421 |
| annual precip. |  | 0.731 | 0.434 |
| coldest month temp. |  |  | 0.487 |
| S pop. | annual precip. | coldest month temp. | coldest month precip. |
| annual temp. | 0.789 | 0.893 | 0.071 |
| annual precip. |  | 0.711 | 0.104 |
| coldest month temp. |  |  | 0.186 |
| N. pop | annual precip. | coldest month temp. | coldest month precip. |
| annual temp. | 0.831 | 0.878 | 0.143 |
| annual precip. |  | 0.720 | 0.301 |
| coldest month temp. |  |  | 0.235 |
| E1 pop. | annual precip. | coldest month temp. | coldest month precip. |
| annual temp. | 0.789 | 0.852 | 0.379 |
| annual precip. |  | 0.679 | 0.447 |
| coldest month temp. |  |  | 0.516 |
| E2 pop. | annual precip. | coldest month temp. | coldest month precip. |
| annual temp. | 0.791 | 0.856 | 0.392 |
| annual precip. |  | 0.680 | 0.511 |
| coldest month temp. |  |  | 0.526 |


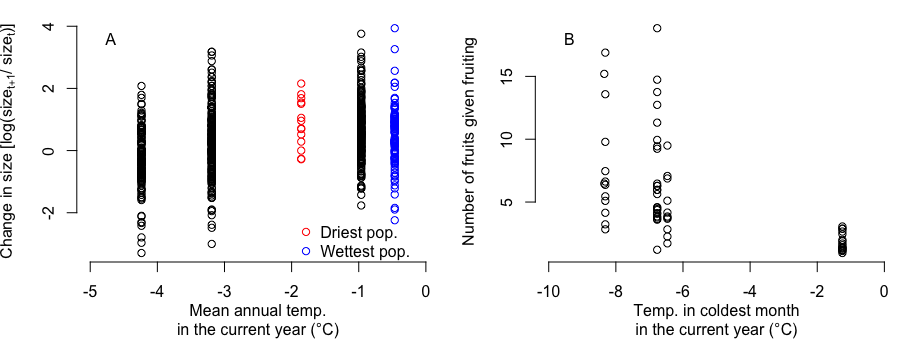


**Figure S1.** Growth and fruiting responses to temperature variation. Analogous to the main text Fig. 3, in A we show data from the driest population and wettest population (driest and wettest are calculated in terms of annual precipitation), in red and blue, respectively.


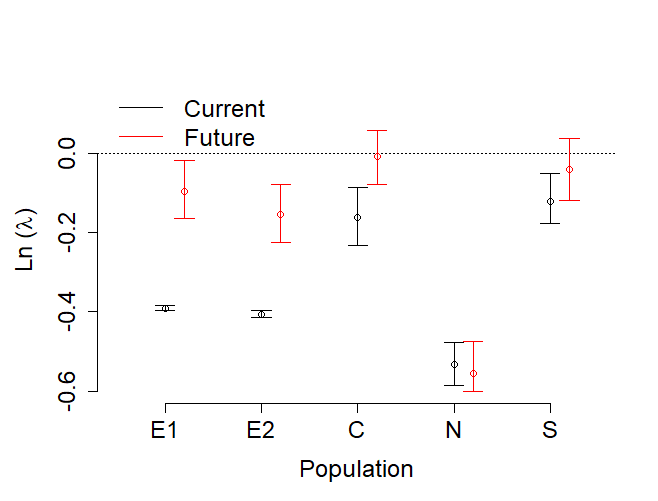


**Figure S2.** The impact of climate change on population growth rates at all populations. Points indicate the mean population growth rate and error bars indicate 95% confidence intervals (calculated across bootstrapped parameter estimates). Populations are arranged along the x-axis by increasing current average annual temperature. Fig. S3 shows GCM-specific results.


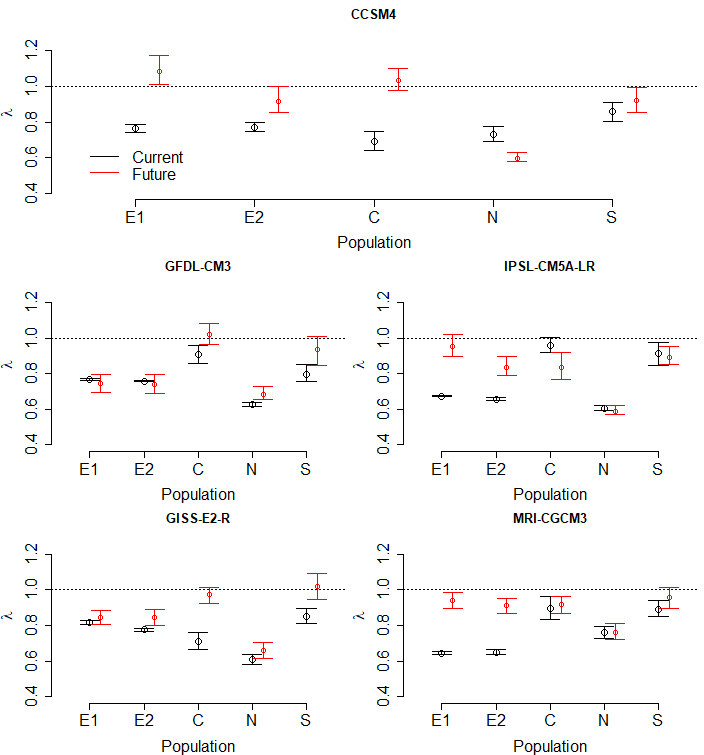


**Figure S3.** The impact of climate change on population growth rates at all populations for each GCM; the name of the GCM is shown in the title of each panel. Points indicate the mean population growth rate and error bars indicate 95% confidence intervals (calculated across bootstrapped parameter estimates). As in other figures, populations are arranged by increasing average annual temperature.


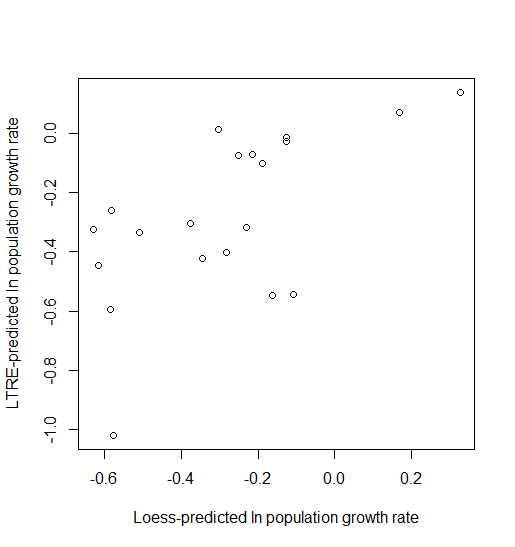


**Fig S4.** Correlation between LTRE-predicted population growth rate and GAM-predicted population growth rate at the mean future climate conditions.


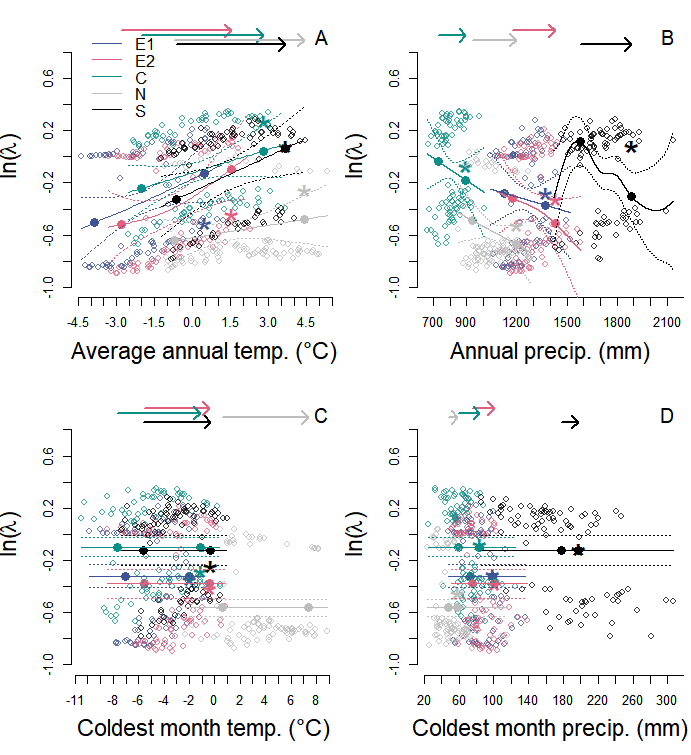
**S5 Fig.** Response of annual population growth rates (λ), to changes in aspects of temperature and precipitation that affect vital rates. Unfilled points represent medians (across bootstrapped regression coefficients) of the annual population growth rates vs. the current and future GCM climate values for each year from 2008 to 2099. Curves represent the fits of GAM predictions, when annual population growth rates are regressed against only annual climate variables. Predictions are calculated when non-focal climate variables are held at their mean values. Arrows on the top of the figures represent the magnitude of climate change at each population; start of the arrow is at the mean climate condition for the current period (2008-2022) and end of the arrow is at the mean climate condition for the future period (2086-2100). Asterisks show the LTRE prediction, as in Fig 7.
